# Enhancing validation of case-control omics signatures through “minimalist” single-subject analysis (N-of-1 trials): proof of concept in sepsis

**DOI:** 10.1093/jamia/ocag061

**Published:** 2026-05-07

**Authors:** Liam S Wilson, Nima Pouladi, Rachel F Nelson, Elizabeth A Middleton, Neal D Tolley, Mahdieh Shabanian, Colleen Kenost, Robert A Campbell, Matthew T Rondina, Yves A Lussier

**Affiliations:** Department of Biomedical Informatics, University of Utah, Salt Lake City, UT 84108, United States; Department of Biomedical Informatics, University of Utah, Salt Lake City, UT 84108, United States; Department of Biomedical Informatics, University of Utah, Salt Lake City, UT 84108, United States; Molecular Medicine Program, University of Utah, Salt Lake City, UT 84112, United States; Department of Internal Medicine, University of Utah, Salt Lake City, UT 84112, United States; Division of Pulmonary and Critical Care Medicine, University of Utah, Salt Lake City, UT 84112, United States; Molecular Medicine Program, University of Utah, Salt Lake City, UT 84112, United States; Department of Biomedical Informatics, University of Utah, Salt Lake City, UT 84108, United States; Department of Biomedical Informatics, University of Utah, Salt Lake City, UT 84108, United States; Molecular Medicine Program, University of Utah, Salt Lake City, UT 84112, United States; Department of Internal Medicine, University of Utah, Salt Lake City, UT 84112, United States; Division of Pulmonary and Critical Care Medicine, University of Utah, Salt Lake City, UT 84112, United States; Department of Pathology, University of Utah, Salt Lake City, UT 84112, United States; Department of Emergency Medicine, Washington University, Saint Louis, MO 63110, United States; Department of Internal Medicine, University of Utah, Salt Lake City, UT 84112, United States; Division of Pulmonary and Critical Care Medicine, University of Utah, Salt Lake City, UT 84112, United States; Department of Pathology, University of Utah, Salt Lake City, UT 84112, United States; Geriatric Research, Education, and Clinical Center (GRECC), G.E. Wahlen VAMC, Salt Lake City, UT 84148, United States; Department of Biomedical Informatics, University of Utah, Salt Lake City, UT 84108, United States; Huntsman Cancer Institute, University of Utah, Salt Lake City, UT 84112, United States; Center for Genomic Medicine, University of Utah, Salt Lake City, UT 84112, United States

**Keywords:** N-of-1 trial, single-subject study, platelet RNA, small clinical trials, sepsis

## Abstract

**Objective:**

To evaluate if a single-subject study (S3) design, utilizing paired transcriptome samples from the same patient (eg, “sepsis” vs “recovered”), can replicate transcriptomic signatures from small case-control studies, addressing challenges in patient accrual for rare or sub-stratified diseases.

**Methods:**

We generated a sepsis gene signature (SGS) comprising 300 differentially expressed genes (DEGs; FDR < 5%) from a human sepsis case-control cohort using general linear models (GLMs). Reproducibility of SGS was assessed through three approaches applied to sub-sampled independent datasets: single-subject analyses (N-of-1-MixEnrich), anticipated to perform better; conventional paired-sample GLM analyses; and a traditional case-control GLM analysis.

**Results:**

SGS reproducibility in GLM analyses was inconsistent at smaller cohort sizes (∼80% reproducibility; *n* = 5) but stabilized at cohort sizes >6. Remarkably, the single-subject-study approach consistently reproduced SGS in each of the 18 subjects individually (100% reproducibility; *n* = 1).

**Discussion:**

Conventional GLMs are not designed for single-subject or small cohort analyses due to their dependence on larger samples to mitigate variable dispersion and human heterogeneity. In contrast, S3 methods enhance statistical power by: reducing multiple testing through gene set aggregation, emphasizing concordant changes in pathway activity rather than exact molecular consistency, and exploiting paired samples from the same individual.

**Conclusion:**

This proof-of-concept demonstrates that S3 designs effectively validate gene expression signatures derived from case-control studies, highlighting their potential in research or clinical trials constrained by small sample sizes. However, further validation and computational simulation are needed to demonstrate scalability to other conditions and sensitivity to validation subject variations from the “average subject” of discovery cohorts.

## Background and significance

Conventional transcriptome methods are designed to uncover and validate common mRNA and pathway signatures across large cohorts of patients.^1^ However, the large sample size requirement of conventional transcriptome analyses has presented a challenge for studying many infrequent clinical disorders where the recruitment of large cohorts is difficult, such as for infrequent diseases or sub-stratified common disorders. Even the conventional designs in isogenic conditions (eg, cell lines, animal models) require at least three biological replicates for applying General Linear Models (**GLMs**^2^^,^^3^). In human studies (heterogeneous conditions), twenty to thirty subjects per group are often required for GLM-based comparisons.

Smaller cohorts are a cardinal feature of infrequent and rare diseases or common diseases with small subgroups. In this context, sepsis is used as a proof-of-concept biological system to test N-of-1 and small-cohort transcriptomic approaches using platelet RNA. Specifically, human platelets contain ∼2.2 fg of total RNA per cell, approximately 400-fold less than peripheral blood mononuclear cells, rendering platelet transcriptomics technically challenging.^4^ Rare and infrequent diseases, collectively, affect ∼8% of the global population and up to 26% of children with disabilities.^5^ Despite over 65,000 diseases, only around ∼3000 are treatable with ∼2500 compounds FDA-approved drugs,^6^ leaving a therapeutic gap exceeding 40 000 diseases that is largely driven by low prevalence and the infeasibility of assembling adequately powered phase 3 clinical trials.^7^

“Single-subject studies” (S3; also known as N-of-1 trials) represent a methodological departure from traditional case-control analyses based on GLMs, offering a patient-centric approach to inference. Unlike traditional approaches that compare different patients, some S3 methodologies infer biological mechanisms by analyzing transcriptome samples drawn from the same individual—either across a time series^6^ or under two discrete conditions, such as sepsis versus recovery, tumor versus adjacent normal tissue, or pre- and post-treatment states. Importantly, gene set-level (eg, pathway) S3 have been shown to unveil altered biomechanisms^7^^,^^8^ more accurately in small cohorts than purely transcript-based ones.^6^^,^^9^ The success of the S3 gene set analyses have not been limited to only theoretical models but have been also corroborated in numerous conditions such as cell and tissue models,^10–12^ as well as retrospectively in predicting cancer survival,^13–16^ circulating tumor cells,^17^ biomarker discovery simulations,^18^ and therapeutic response.^13^^,^^14^ Previously published S3 designs^19^ have overall demonstrated that gene set-level transcriptome signals improve statistical power by: (1) reducing the total features tested and (2) relaxing the requirement of within-cohort concordance of each differentially expressed transcript.

The aim of this study is to design a method for validating transcriptome signatures (derived from case-control cohort studies) via a single-subject study. Eventually, this could enable clinical trials in smaller cohorts, which would lower the cost threshold for studying of infrequent diseases or sub-stratified frequent disorders.

## Materials and methods

**Figure 1.**
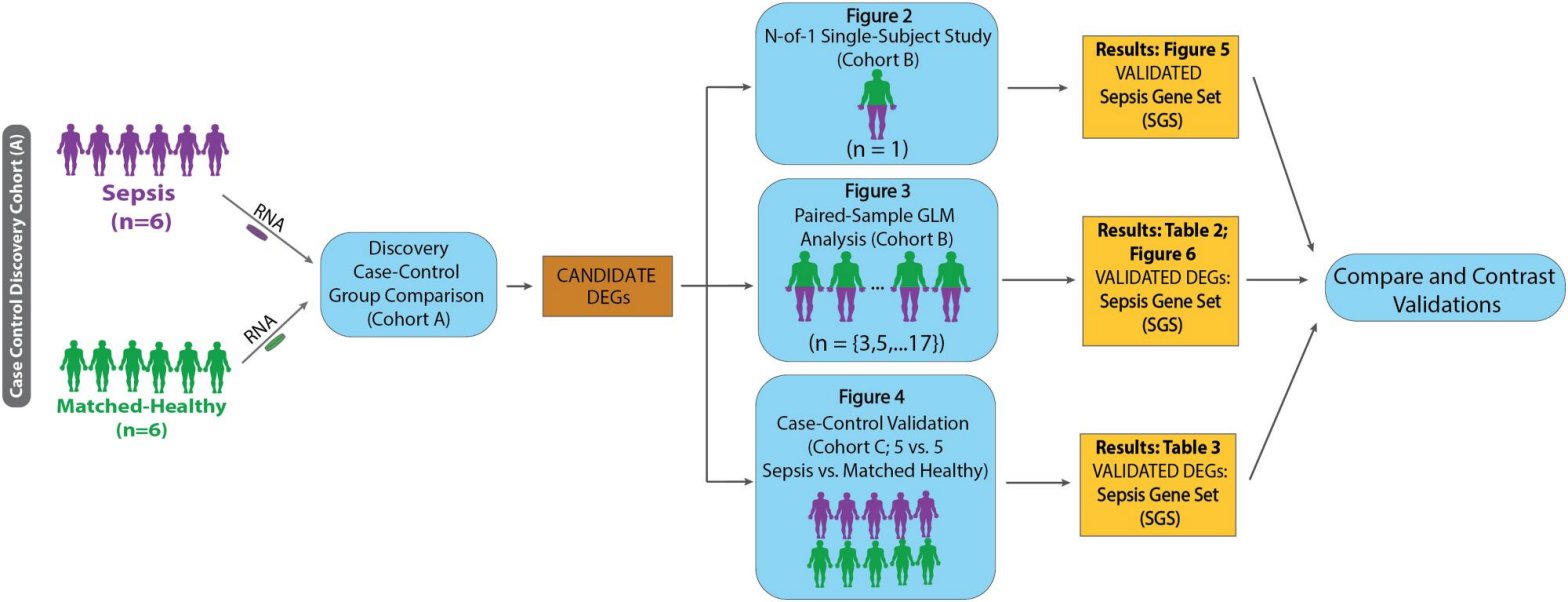
Overview of methods. A sepsis gene signature (SGS) was derived from a case-control comparison of platelet transcriptomes in 6 sepsis patients and 6 matched healthy controls. SGS reproducibility was evaluated using three validation strategies: (1) single-subject (N-of-1) analyses applying mixture models to paired longitudinal samples to identify altered transcripts by posterior probability; (2) paired-sample GLM analyses with repeated resampling of the same longitudinal cohort across varying sample sizes; and (3) an independent case-control cohort of 5 sepsis patients and 5 matched controls. Here, differentially expressed genes (DEGs) identified by cross-subject analyses can be validated by cross-subject cohorts, whereas gene-set-level validation can occur in all three types of validations including a single-subject study.

**Figure 2.**
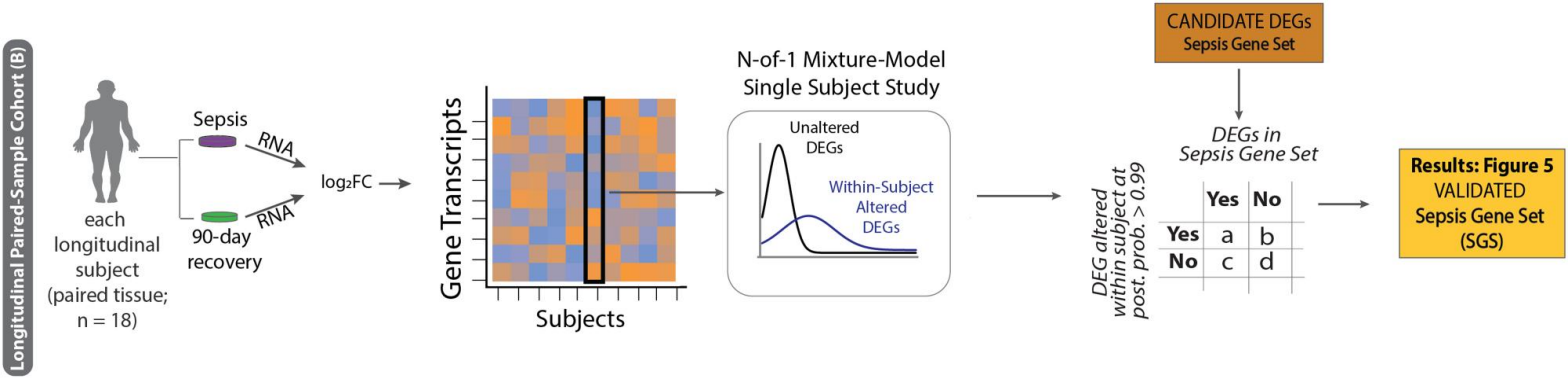
N-of-1 single-subject-study. A single-subject analytical method, N-of-1-MixEnrich, was utilized to show whether the candidate DEGs (SGS) could be replicated in paired sample design of cohort B. Specifically, in each patient separately, a statistical mixture model composed of two distributions was fitted to transcript log2-fold change to discover altered transcripts. Then, the enrichment of genes with altered expression in each subject, within SGS, is calculated as an odds ratio from point estimate of Fisher’s Exact Test with its 95% confidence interval (see Supplementary Material 2).

**Figure 3.**
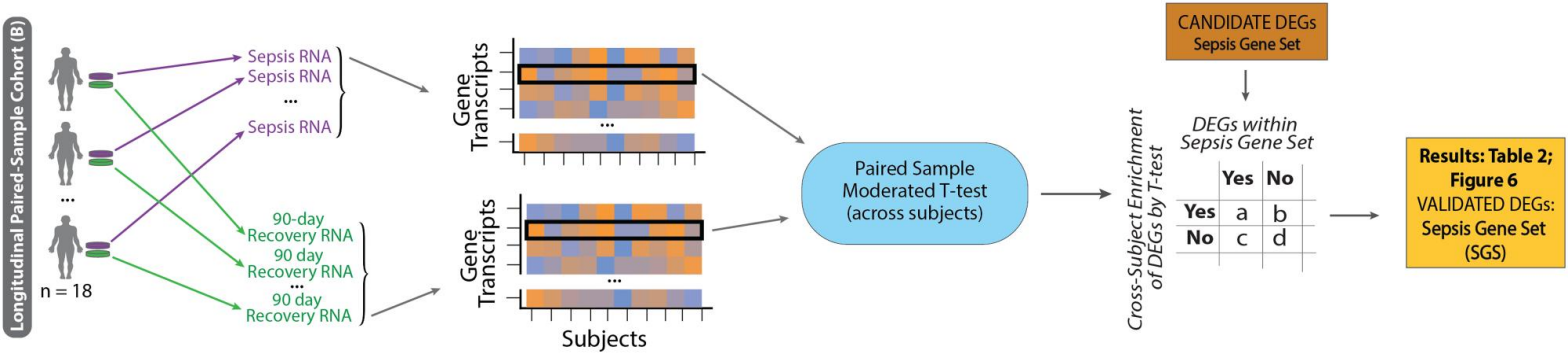
Cohort-size dependence paired-sample *t*-test analysis. Subjects were down-sampled into 10-subject cohorts sized *n* = 3 to *n* = 17 and analyzed with paired *t*-tests. Overlap of DEGs in SGS were analyzed with Fisher’s exact test and results were summarized as a table of ORs (Table 3) and in boxplots (Figure 6).

**Figure 4.**
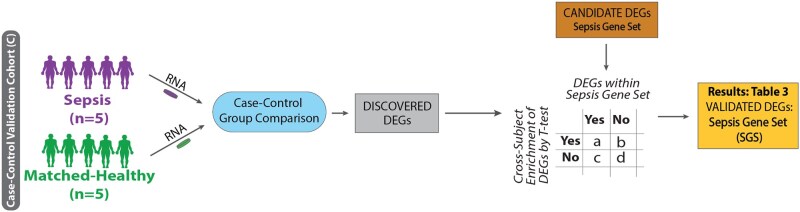
Independent case-control cohort analysis. DEGs were identified in cohort C using a moderated *t*-test, and SGS enrichment was assessed using Fisher’s exact test (*P* < .05). Results were summarized as a table of odds ratios (Table 4).

*Overview of the study design and methods* (Figure 1). An initial set of candidates differentially expressed genes (DEGs), termed the Sepsis Gene Signature (SGS), was identified through a case-control comparison between 6 sepsis patients and 6 matched healthy controls. This SGS was subsequently validated using three independent approaches: (1) an N-of-1 single-subject study using posterior probabilities from a mixture model applied to a new longitudinal dataset (Figure 2), (2) a paired-sample General Linear Model (GLM) analysis with repeated resampling also applied to a new longitudinal dataset (Figure 3), and (3) an independent, previously published case-control dataset comprising 5 sepsis patients and 5 matched controls (Figure 4). Validation performance was assessed using Fisher’s exact test to evaluate overrepresentation of either altered transcripts or DEGs within the SGS. Notably, the term DEGs is reserved for genes identified through cross-subject analyses (eg, paired sample *t*-test, GLM, or moderated *t*-test), whereas “altered transcripts” refers specifically to those identified in single-subject studies using posterior probability thresholds in mixture models. The results of the single subject studies are compared to those obtained by resampling the longitudinal dataset at different subject cohort sizes and conducting conventional GLM analyses. Finally, while cross-subject methods can validate both the DEGs and the gene set, the single subject study is limited to validation of the gene set by design.

Abbreviations used in this manuscript are defined in Supplementary Material 1.

### Study populations

We used three independent cohorts in our study (Table 1). Specifically, we utilized data from a previously published platelet RNA-seq sepsis case-control cohort (6 sepsis patients and 6 matched healthy controls^21^) to derive a platelet-based Sepsis Gene Signature (SGS) (Cohort A, Table 1). We used a novel longitudinal cohort (Cohort B; Methods Tables 1 and 2), which included patients with confirmed sepsis at ICU admission and follow-up samples collected 90 days post-recovery. Platelet RNA samples were obtained at both timepoints from each of the 18 patients in this cohort. We finally used another independent case-control dataset (Cohort C, Table 1) for testing reproducibility of the SGS in the same cohort design (5 sepsis patients and 5 matched controls^20^).

**Table 1.** Description of datasets used for obtaining and validating SGS.

	Discovery	Validation	
Reference	Campbell et al 2022	Novel	Middelton et al 2019
Cohort type	Case-control	Longitudinal	Case-control
Cohort label	A	B	C
Tissue (*)	Platelet RNA	Platelet RNA	Platelet RNA
Subjects counts	6 vs 6 (matched) sepsis cases vs healthy controls (**)	18 sepsis cases	5 vs 5 (matched) sepsis cases vs healthy controls (*)

Sepsis was defined using the consensus criteria [20]. For validation purpose we utilized two more cohorts: (1) a paired sample longitudinal cohorts (*n* = 18 unique patients with their samples taken during contraction of sepsis and then after recovery), and (2) a distinct matched case-control (*n* = 5 vs *n* = 5). The selection of platelet RNA as a biospecimen (*) is supported by the stability of transcripts in anucleate platelets over their ∼10-day lifespan, during which gene expression remains unaltered following release from bone marrow megakaryocytes. Healthy donor controls (**) were matched at enrollment for age and sex. The cohorts were labeled as A, B, C for brevity.

**Table 2.** Demographic and comorbidity characteristics of the novel longitudinal cohort (Cohort B), in which each subject contributed paired platelet RNA samples collected during acute sepsis and again 90 days post-recovery.

Clinical characteristic	Value
Sex*	Female [*n* = 9], Male [*n* = 9]
Mechanical Ventilation*	3 (Yes) 15 (No)
SHOCK *	10 (Yes) 8 (No)
Age (yrs)	65.00 [*n* = 18] (55.50–69.75) (14.25)
Height (cm)	172.70 [*n* = 18] (168.25–177.18) (8.93)
Weight (kg)	94.25 [*n* = 18] (84.19–104.98) (20.79)
BMI*	30.94 [*n* = 18] (28.21–37.18) (8.97)
SOFA Score	6.50 [*n* = 18] (4.25–8.00) (3.75)
APACHE II Score	30.50 [*n* = 18] (22.25–78.50) (56.25)
Length of ICU Stay (hours)	66.52 [*n* = 18] (40.49–90.29) (49.80)
WBC (White Blood Cell; count/µL)	13.75 [*n* = 18] (9.80–18.08) (8.28)
Hemoglobin (g/dL)	12.25 [*n* = 18] (10.70–14.77) (4.07)
Creatinine (mg/dL)	1.67 [*n* = 18] (1.17–2.77) (1.60)
Platelets (count/µL)	117.00 [*n* = 17] (27.00–232.00) (205.00)

This cohort is used as a validation dataset in methods described in Figures 2 and 3. The RNA-seq data generated at the University of Utah will be deposited in the NCBI dbGaP repository and made available under accession number phsxxxxx.vx.px (to be provided upon manuscript revision and acceptance). Median [*n* = subjects’ count]; (Quartile 2 - Quartile 3) (Inter-Quartile Range); * Categorical.

SOFA, Sequential Organ Failure Assessment (A metric of acute morbidity of critical illness [22]).

APACHE II: (A measure to evaluate severity of sepsis disease [23]).

BMI, Body Mass Index; SHOCK, septic shock and vasopressors prescribed (Yes/No).

Details on clinical phenotyping, sample preparation, and RNA library construction have been previously described.^20^^,^^21^ These case-control datasets were obtained through author correspondence (University of Utah co-authors) and are not publicly available. Patients were prospectively enrolled within 48-72 hours of ICU admission for severe sepsis or septic shock, as defined by consensus criteria.^24^ Matched healthy controls in both the discovery and validation cohorts were screened to exclude individuals with bleeding disorders, liver or kidney disease, cancer, recent surgery or thrombotic events (within 3 months), and those receiving antiplatelet or anticoagulant therapy.

### Statistical analysis

Our analytical pipeline comprises four analyses. First, a conventional case-control cohort (6 sepsis cases vs 6 matched healthy controls^21^) comparison by which we derive SGS (Figure 1**)**, which serves as a case-cohort discovery study that awaits to be reproduced in a validation study. We propose three types of validation study designs that are compared:

- (i) A set of single-subject studies (S3 or N-of-1; Figure 2) in our new longitudinal cohort (Table 1), each of which is used to validate the SGS using enrichment statistics in a single subject.
- (ii) Paired *t*-test statistics (Figure 3) in our new longitudinal cohort (Table 1) analysis across sub-cohorts of varying size; again, followed by enrichment statistics of the discovered DEGs in the SGS.
- (iii) A conventional case-control cohort comparison (*t*-test; Figure 4) in a separate independent validation cohort from the discovery one (5 matched healthy vs. 5 sepsis subjects^20^) also followed by enrichment analysis of discovered DEGs in SGS.

#### Processing and normalization of raw RNA count value

We processed the case-control discovery cohort using the NOISeq R package^25^^,^^26^ and filtered out the transcripts with low count, using cpm <10 with method one (default parameters). This procedure resulted in 3227 unique transcripts in the discovery cohort. Subsequently, we performed NOISeq TMM (Trimmed Mean of *M*-values)^26^ normalization for downstream statistical analysis. We applied similar processing and normalization criteria to each of the raw RNA counts of the case-control data sets and the longitudinal validation cohort separately and found 5003, and 2536 TMM normalized transcripts, respectively.

#### Conventional cohort comparison to discover DEGs and a sepsis gene signature (Figure 1)

*a) Discover platelet-derived differentially expressed genes between sepsis subjects vs matched control subjects in order to create SGS.* We applied the “voomWithQualityWeights” function^27–29^ from the *limma* package on each case-control cohort separately to enable GLM analysis.^30^^,^^31^ We utilized the moderated *t*-test implemented in *limma* and found **SGS** given (1) absolute log2 fold change greater than 1.5 and (2) BH-corrected *P*-value (FDR) < .05^32^ in the discovery cohort.

#### Evaluate the ability of a single-subject-study to validate DEGs amongst paired-samples in a longitudinal cohort

The N-of-1-MixEnrich is an analytical method^14^ employed in this study to identify deregulated gene sets in single-subject study designs (Figure 2). Briefly, it applies statistical mixture modeling^33^ to the absolute value of the log_2_ fold change (**FC**) expression of each transcript of a subject from expression taken in two conditions (eg, treated vs untreated, disease vs healthy). It then provides a posterior probability for each FC that enables grouping as either (1) altered expression or (2) unaltered (including up- and down-regulated at desired posterior probability threshold; the higher, the more stringent). Next, it evaluates the statistical significance of the overlap of a desired a priori known gene set (here, SGS) with the list of altered transcripts using a contingency table and deriving the odds ratio (defined as an effect size) whose statistical significance is evaluated with Fisher’s Exact Test (FET; *P*-value < .05). For each of the 18 patients in the longitudinal cohort, we applied the N-of-1-MixEnrich method^14^ to their absolute-value log2 fold change transcriptome profiles. We identified altered transcripts with a stringent posterior probability cutoff of >0.99, ensuring a high-confidence selection of differentially expressed genes. This optimal threshold, validated in prior studies,^34^ was chosen to minimize false positives and increase the specificity of findings. We then used Fisher’s Exact Test and evaluated the statistical significance of the overlap of each patient’s identified altered transcripts and the SGS (FET *P* < .05).

#### Evaluate cohort size dependency of the paired sample GLM (t-test) analysis in rediscovering the SGS in a longitudinal cohort using decreasing subject counts resampling

To assess sample-size dependence of rediscovering SGS under paired sample conditions, we created 10 randomized cohorts for various counts of patients from our new longitudinal cohort (Tables 1 and 2), ranging from 3 to 17 with increments of two, without replacement (Figure 3). We used a moderated *t*-test for paired-design experiments (*limma*; FDR < 0.05). For each set of findings, we then evaluated the overlap of DEGs (for each randomization; |log2FC| > 1.5) with all transcripts found as SGS with Fisher’s exact test (*P*-value < .05). We presented the findings with boxplots indicating successful or failed recapitulation of SGS in each randomized cohort.

#### Evaluate the reproducibility of the case-control-discovered DEGs in another case-control cohort

We then evaluated the reproducibility and statistical significance of SGS in a validation cohort (Cohort C) composed of 5 sepsis subjects and 5 matched healthy controls,^20^ distinct from cohort A (Figure 4). Specifically, we made use of a moderated *t*-test in this distinct case-control validation cohort to discover DEGs (*limma*; FDR <0.05), and then proceeded to evaluate the statistical significance of enrichment amongst DEGs discovered in this validation cohort within SGS with Fisher’s exact test (*P*-value < .05). We presented the findings with a table of odds ratios.

## Results

To assess the presence of a global transcriptomic signal associated with sepsis, we analyzed RNA-seq data from three patient cohorts (Table 1). Prior studies established the comparability of clinical characteristics within each case-control cohorts (A and C; Table 1), and we similarly assessed key clinical and laboratory variables in the longitudinal cohort B (Tables 1 and 2). Unsupervised principal component analysis (PCA; data not shown) of transcriptomic profiles demonstrated clear segregation of sepsis and healthy samples in both the discovery and validation case-control cohorts, with the first principal component explaining 46% and 45% of the variance in cohorts A and C, respectively. Similarly, unsupervised clustering in cohort B confirms segregation of the sepsis states and paired recovered states along with the first and the second principal components with 57% and 29% of the variability, respectively. No batch effect was observed in these analyses.

In the cohort A, we then identified 300 DEGs (153 up-regulated and 147 down-regulated DEGs in cases; FDR < 5%, |log_2_FC|>1.5) that distinguish sepsis from healthy/recovery states in platelet RNAs. We then proceeded to evaluate the capability of three distinct statistical methods to reproduce the SGS in our validation cohorts.

**Figure 5.**
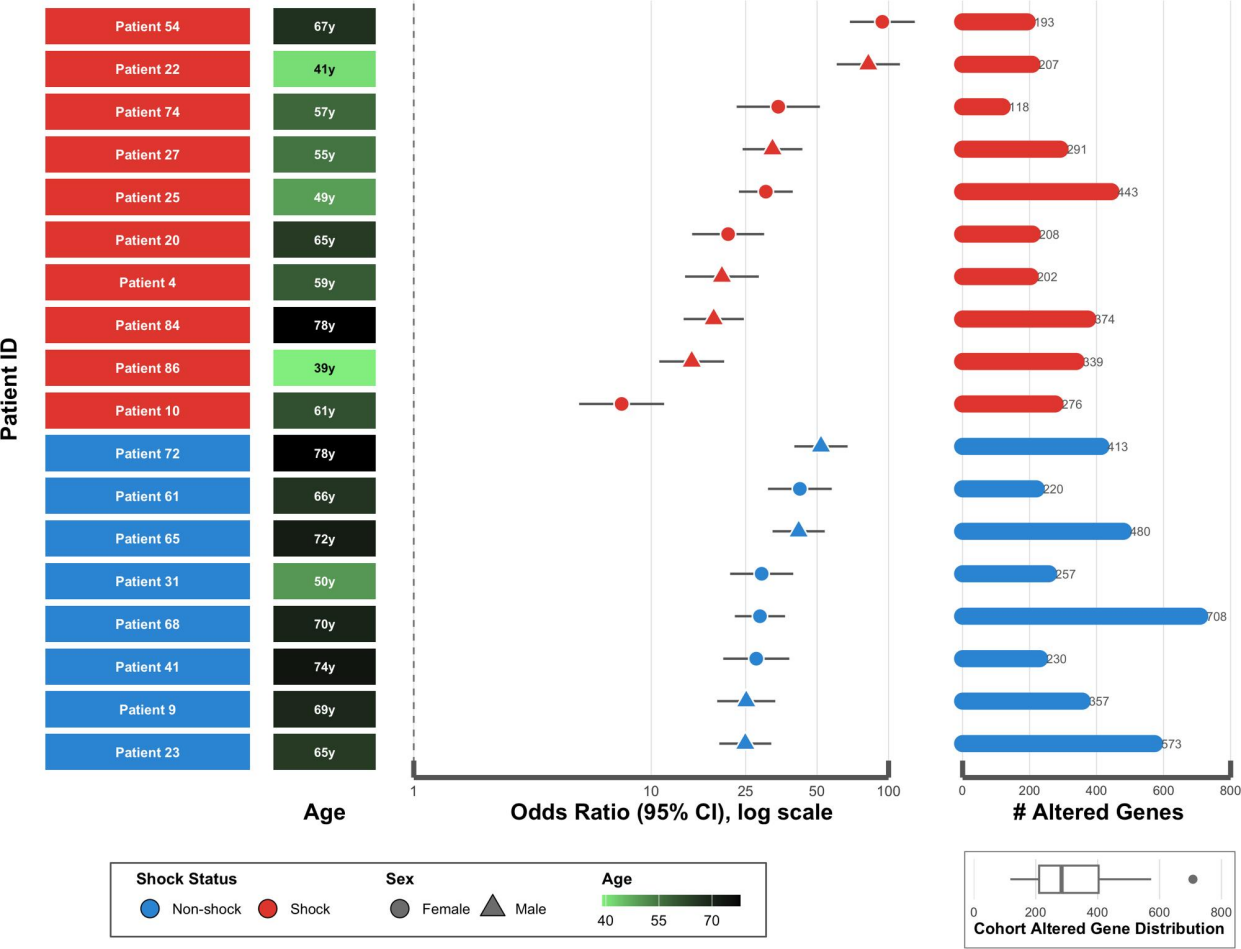
Each single-subject study validates the sepsis gene signature. Each row represents one independent single-subject study from the longitudinal cohort (Cohort B; Table 2). Paired platelet RNA-seq samples collected during sepsis and recovery were analyzed together within each individual and separately from other subjects. Together, the panels illustrate consistent SGS enrichment across all 18 single-subject studies, alongside substantial inter-individual variability in effect size and number of altered transcripts. *Left panel*: Patient identifiers and age at enrollment. *Middle panel*: Forest plot showing the odds ratio (OR) and 95% confidence interval for enrichment of SGS genes among altered transcripts identified within each subject using the N-of-1-MixEnrich method. Odds ratios were computed from Fisher’s exact test using a 2 × 2 contingency table comparing altered versus unaltered transcripts by SGS membership and are displayed on a logarithmic scale (see Supplementary Material 2). *Right panel*: Number of altered transcripts identified per subject at a posterior probability threshold >0.99. *Bottom Right*: The inset boxplot summarizes the distribution of altered transcript counts across the cohort. Red vs blue color indicates shock status; symbol shape denotes sex; age is shown by green color intensity.

We then evaluated the reproducibility of SGS in cohort B in each subject. As shown in Figure 5, each of the 18 single-subject studies exhibited substantial enrichment (Odds Ratio) and statistical significance of SGS, providing robust within-subject corroboration of the transcriptomic signal originally discovered in the case-control cohort. To control for false positives, we applied N-of-1-MixEnrich to 999 random gene sets matched in size to the SGS, evaluating significance (posterior probability > 0.99, FDR < 0.05) in each of the 18 subjects individually. As expected, none of the random sets yielded significant enrichment in any subject.

**Figure 6.**
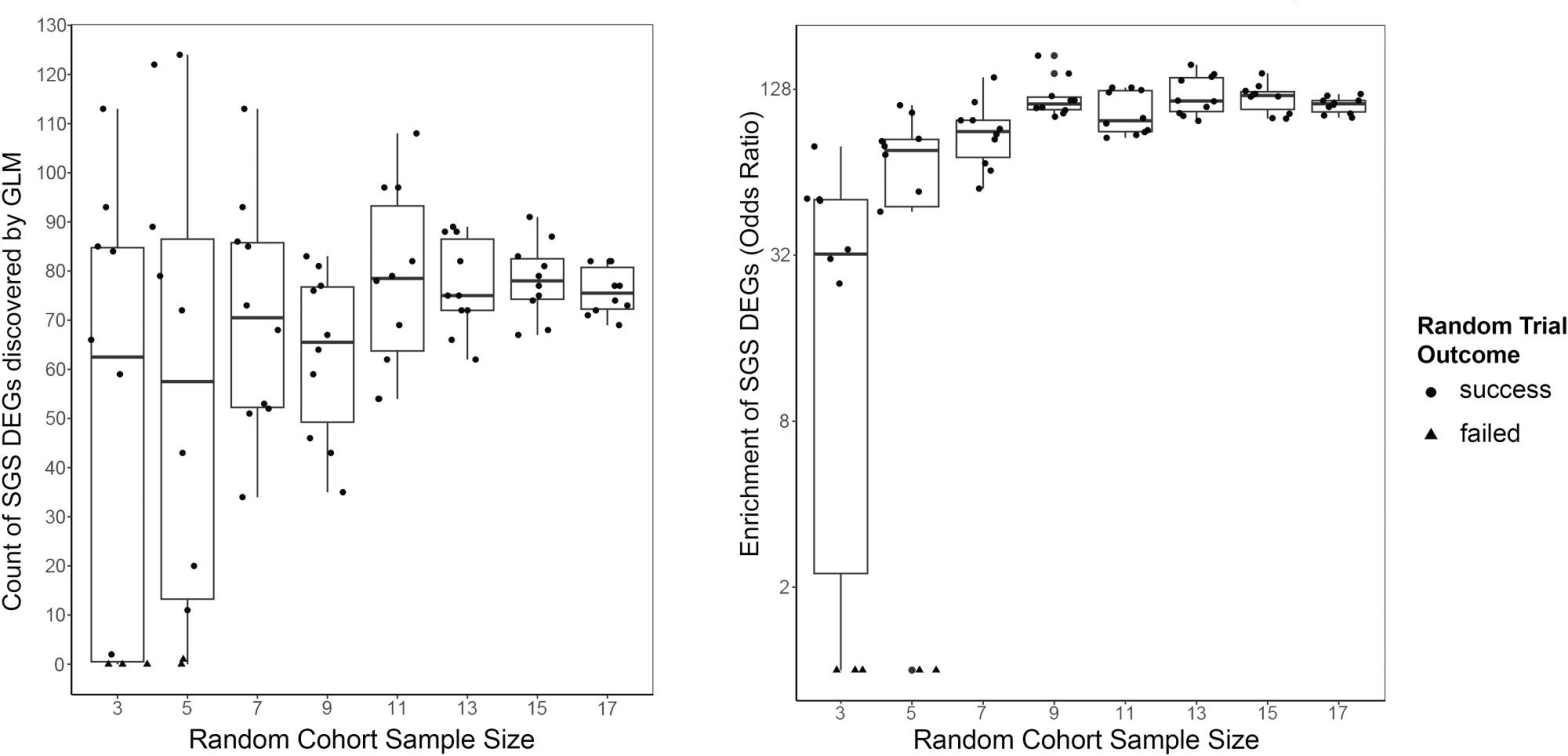
Paired *t*-test fail to reproduce the Sepsis Gene Signature (SGS) below a cohort size threshold (*n* = 7) in longitudinal studies. Boxplots display the distribution of odds ratios (left panel) and counts of significantly overlapping SGS genes among DEGs (right panel) identified using a paired *t*-test applied to randomly sampled cohorts of varying sizes (*x*-axis) from the longitudinal study (Table 1). DEGs were prioritized using |log_2_FC| ≥ 1.5 and FDR ≤ 0.05. Overlap with SGS genes was evaluated using Fisher’s exact test (*P* < .05).

Next, we evaluated how conventional paired *t*-test statistics perform in comparison. Specifically, how many additional subjects would be needed to validate SGS in cohort B (Table 1), in which each individual subject (Figure 5) had already confirmed the SGS through single-subject analysis. As shown in Table 3, while the number of DEGs identified at a fixed FDR < 5% is highly sensitive to the applied fold change (FC) threshold, the SGS is still reproduced when all 18 subjects are included. We then conducted random sampling at distinct cohort sizes in Figure 6. When cohort size was fewer than 7 subjects, GLM-based analyses consistently failed to reproduce SGS DEGs. Cohort sizes under 15 also exhibited greater dispersion in both odds ratios and the number of overlapping DEGs. These findings highlight a lower limit for cohort size in GLM reproducibility and reinforce the utility for the proposed single-subject validation approaches in transcriptomic studies of infrequent diseases where patient accrual is rate limiting.

**Table 3.** Paired samples *t*-test analyses validate the SGS in all fold change thresholds.

Validation cohort	Cohort size	| log_2_FC |	**Prioritized DEGs** * **(Count)**	SGS transcripts among prioritized DEGs	Odds ratio
Longitudinal	Sepsis (*n* = 18) vs Recovered (*n* = 18)	0	1643	194	22.9 #
		0.5	1218	190	31.2 #
		1	420	141	60.1 #
		1.5	129	74	112.4 #

18 subjects with paired samples.

*At FDR < .05.

# All *P*-values <10^−20^ (Fisher’s exact test).

Finally, we performed conventional validation by applying the same case-control design to an independent cohort C.^20^ As shown in Table 4, although the number of DEGs at a fixed FDR < 5% is highly sensitive to the fold change threshold, the SGS remains reproducible using all 5 sepsis cases and 5 matched controls.

**Table 4.** Case-control *t*-test analyses in an independent cohort C validate the SGS in all fold change thresholds.

Validation cohort	Cohort size	| log_2_FC |	**Prioritized DEGs** * **(count)**	SGS transcript among prioritized DEGs	Odds ratio
Case-Control	Sepsis (n = 5) vs Healthy (n = 5)	0	1469	175	10.3 #
		0.5	1455	174	10.1 #
		1	792	154	14.9 #
		1.5	347	119	23.3 #

*Significant at FDR < .05.

# All *P*-values <10^−20^ (Fisher’s exact test).

## Discussion

This proof-of-concept study demonstrates that a single-subject study can validate a gene set of differentially expressed genes derived from traditional case-control transcriptomic analyses using paired samples from an individual patient. Specifically, a sepsis gene signature identified in an independent case-control cohort was reproducibly enriched within each subject of a longitudinal cohort when comparing paired sepsis and recovery samples. This result indicates that validation of case-control transcriptomic signatures need not rely on assembling a second, independent cohort, an advantage in settings where patient accrual is constrained by disease rarity or clinical sub-stratification.

In this study, the single-subject framework treated each patient as their own control in a longitudinal design, contrasting platelet transcriptomes collected during acute sepsis with those obtained following recovery. To discover altered transcripts in each subject, we applied the N-of-1-pathways-MixEnrich,^14^ method for single-subject transcriptome analysis, as it requires only two samples per subject (one per condition) and has demonstrated improved performance relative to alternative single-subject approaches, including N-of-1-pathways Wilcoxon and Mahalanobis distance–based methods.^6^^,^^11^^,^^15^^,^^17^^,^^18^ Altered transcripts identified within individual subjects were then evaluated for enrichment within the SGS discovered in the case-control cohort, providing a statistically interpretable effect size and significance at the gene-set level. As illustrated in Figure 5, SGS validation was achieved independently within each subject. Across all 18 subjects, this approach consistently reproduced the SGS, despite substantial inter-individual variability in the magnitude of transcriptomic response.

In contrast, conventional paired-sample general linear model analyses applied to the same longitudinal dataset required substantially larger cohort sizes to achieve stable SGS reproduction, failing consistently below approximately seven subjects and remaining variable at intermediate sample sizes (Figure 6). Together, these results highlight a fundamental distinction between cohort-level inference, which depends on averaging across heterogeneous individuals to suppress variance, and single-subject inference, which exploits within-individual isogenic pairing to reduce biological and technical variability at the source.

The inter-subject dispersion of odds ratios observed in Figure 5 indicates that, while SGS enrichment was consistently detected, the magnitude of transcriptomic response to sepsis varied considerably between individuals. This variability was not explained by available clinical covariates, suggesting that phenotypic similarity to the discovery cohort may influence single-subject validation performance.

### Limitations

By design, paired-sample single-subject studies using mixture models produce posterior probabilities for altered transcripts, which are not directly comparable to FDR-based DEG identification across subjects. Consequently, the single-subject approach we propose enables validation at the gene set level, quantified by an effect size (odds ratio) and *P*-value for the entire set, but does not support inference on individual transcripts. In this study, inference further depends on fixed posterior-probability thresholds, which may limit sensitivity and specificity relative to alternative thresholding strategies within the same mixture-model framework. Alternative single-subject designs incorporating repeated measures over time may, in principle, enable transcript-level inference for a limited number of genes; however, such multivariate longitudinal approaches have not yet been validated at transcriptome scale.

In addition, blood samples were collected within a 48-72 hour window following ICU admission, introducing temporal biological heterogeneity given the rapid evolution of sepsis-associated transcriptomic responses. Importantly, such heterogeneity would be expected to attenuate rather than enhance detection of a consistent disease signal. This variability is mitigated by robust detection of sepsis from platelet RNA, indicating resilience to differences in sampling time. Indeed, circulating leukocytes persist in blood for hours to a few days and actively remodel their transcriptomes through signal-responsive nuclei, whereas platelets circulate for ∼7-10 days^35^ as anucleate cells carrying a fixed RNA payload, resulting in fundamentally different temporal sensitivities: platelet transcriptomes buffer, whereas leukocyte transcriptomes amplify, sampling-time heterogeneity.

With respect to cohort composition, the single-subject validation cohort excluded patients receiving antiplatelet or anticoagulant therapy, which may limit generalizability to higher-risk septic populations in whom such therapies are common.

Data access to cohort previously published and current cohort demographics is restricted under the IRB protocol, precluding re-contact of subjects to confirm or expand demographic characteristics; moreover, although prior studies report matched case-control cohorts, demographic details are incompletely reported and inter-cohort comparability cannot be fully assessed, a limitation additionally mitigated by successful replication of the signature across both case-control and longitudinal cohorts. Furthermore, while survivor bias affects the longitudinal single-subject cohort due to required follow-up sampling, this bias is not present in the discovery and validation case-control cohorts, which in principle should have outperformed the single-subject analyses given larger sample sizes and broader severity coverage; thus suggesting that the observed robustness of S3 performance is attributable to the improved informatics/analytic model rather than cohort composition.

### Future studies

Future studies could leverage the inter-subject variability observed in SGS enrichment to develop principled subject selection strategies, such as phenotypic distance metrics that identify individuals most representative of the discovery population or, alternatively, mechanistic outliers. Such approaches would enable systematic evaluation of S3 robustness under increasing biological heterogeneity and inform inclusion criteria for single-subject validation studies.

In parallel, we are exploring extensions of the S3 framework to emerging high-resolution transcriptomic modalities. Single-cell transcriptomics, which capture expression variability across cell types within a single sample, may enable differential expression analyses in paired-sample designs that approximate multi-subject case-control comparisons. A similar rationale applies to spatial transcriptomics, where diseased tissue samples include adjacent unaffected margins that can serve as within-sample comparators.

Future research should also explore the integration of S3 methodologies into clinical trials, with the dual aims of reducing trial costs and enabling finer-grained molecular sub-stratification in both common and rare diseases. Paired-sample designs, while well suited to rare-disease research, remain unconventional and therefore lack readily available historical datasets, underscoring the need for prospective study design.

The proposed approach is conceptually extensible to other dynamic ‘omics layers, including metabolomics, methylomics, and proteomics. To support broader adoption, large-scale simulations will be essential to characterize the statistical power of S3 designs across varying gene set sizes and levels of biological heterogeneity. In addition, sources of inter-subject variability in gene set enrichment, beyond demographic and clinical variables, warrant further investigation.

Prior work on Inter-N-of-1 designs has shown that as few as three single-subject studies, followed by meta-analysis, can reproduce classical case-control findings across multiple historical datasets and large-scale simulations.^34^ Incorporation of additional phenotypes, including treatment exposures and comorbidities, may facilitate the construction of *a priori* phenotypic distance metrics that assess whether a subject aligns sufficiently with a reference population to serve as a valid comparator, or instead constitutes a mechanistic outlier. These metrics could inform single subject study inclusion criteria and improve the selection of single-subject profiles best suited for validation studies.

## Conclusion

This proof-of-concept study shows that single-subject (S3) designs, “one patient at a time,” can validate gene expression signatures derived from traditional case-control transcriptomic analyses using paired samples from individual patients. The analyses were performed on newly generated, previously unpublished datasets, thereby minimizing dataset selection bias and strengthening the internal validity of the findings. Because the approach operates on quantitative molecular measurements and gene-set–level aggregation rather than cohort-level inference, it is conceptually extensible to other high-dimensional ‘omics modalities with similar properties, including proteomics, metabolomics, and epigenomic assays such as DNA methylation. Clinical trials involving multimolecular biomarkers may be well suited to this approach, especially in settings constrained by limited cohort sizes.

## Supplementary Material

ocag061_Supplementary_Data

## Data Availability

Discovery (A) and validation cohort (C) RNAseq data are available through Campbell 2022 and Middleton 2019; the novel longitudinal cohort RNAseq expression, associated analytical pipelines, and software code are available at GitHub: https://github.com/wilsonliam/case-control-single-subject-validation.
