# Supplementary material for: Enhancing validation of case-control omics signatures through “minimalist” single-subject analysis (N-of-1 trials): proof of concept in sepsis
Source: J Am Med Inform Assoc. 2026 May 7;33(7):1293–303. doi: 10.1093/jamia/ocag061 (PMC13317961; doi:10.1093/jamia/ocag061)
Supplement: ocag061_Supplementary_Data [file ocag061_supplementary_data.zip › jamia-sss-valid-supp-1.docx]

**Supplementary Material 1. Abbreviations**

| **Abbreviations** | **Full Form** |
| --- | --- |
| APACHE II | Acute Physiology and Chronic Health Evaluation II (sepsis severity evaluation) |
| BH | Benjamini-Hochberg (FDR) correction |
| BMI | Body Mass Index |
| cpm | Counts per million |
| DEG(s) | Differentially Expressed Gene(s) |
| FC | Fold Change |
| FDA | Food and Drug Administration |
| FDR | False Discovery Rate |
| FET | Fisher's Exact Test |
| GEO | Gene Expression Omnibus |
| GLM(s) | General Linear Model(s) |
| ICU | Intensive Care Unit |
| MD | Mahalanobis Distance |
| mRNA | messenger RNA |
| N-of-1 | Single-subject study |
| OR | Odds ratio |
| PCA | Principal Component Analysis |
| RNA-seq | RNA sequencing |
| S3 | Single-Subject Study |
| SD | Standard deviation |
| SGS | Sepsis Gene Signature |
| SNOMED | Systematized Nomenclature of Medicine |
| SOFA | Sequential Organ Failure Assessment (sepsis severity evaluation) |
| TMM | Trimmed Mean of M-values (normalization) |
| WBC | White Blood Cell |
