# Supplementary material for: Enhancing validation of case-control omics signatures through “minimalist” single-subject analysis (N-of-1 trials): proof of concept in sepsis
Source: J Am Med Inform Assoc. 2026 May 7;33(7):1293–303. doi: 10.1093/jamia/ocag061 (PMC13317961; doi:10.1093/jamia/ocag061)
Supplement: ocag061_Supplementary_Data [file ocag061_supplementary_data.zip › jamia-sss-valid-supp-2.docx]

**Supplementary Material 2. Computation of Odds Ratio and its Variance**

We computed the error bars that represent a 95% standard deviation around the odds ratio (OR). The OR and its variance were computed from a 2×2 contingency table cross-classifying altered transcripts (posterior probability > 0.99) by Sepsis Gene Signature (SGS) status for each patient (**Supplementary Table A**, **Equations 1 and 2**). To account for small-sample bias, a continuity correction of 0.5 was added to each cell of the contingency table (Agresti et al 2011)

**Supplementary Table A.** Calculation of the odds ratio in a single-subject study using altered transcripts identified by mixture models applied to expression fold changes between two samples from the same subject: one obtained during sepsis and one obtained in the non-sepsis condition.

|  | Altered Transcript | Not Altered Transcript |
| --- | --- | --- |
| in SGS | a | c |
| Not in SGS | b | d |

**Equations 1 and 2**

$$OR=\frac{\left( a+\frac{1}{2} \right)\left( d+\frac{1}{2} \right)}{\left( c+\frac{1}{2} \right)\left( d+\frac{1}{2} \right)}$$

$$Var\left( OR \right)=\frac{1}{\left( a+\frac{1}{2} \right)}+\frac{1}{\left( b+\frac{1}{2} \right)}+\frac{1}{\left( c+\frac{1}{2} \right)}+\frac{1}{\left( d+\frac{1}{2} \right)}$$
